# Should we build single-cell lineage trees from gene expression data?

**DOI:** 10.1093/genetics/iyag187

**Published:** 2026-07-18

**Authors:** Nicola Mulberry, Tanja Stadler

**Affiliations:** Department of Biosystems Science & Engineering, ETH Zürich, Basel 4058, Switzerland; Swiss Institute of Bioinformatics, Lausanne 1015, Switzerland; Department of Biosystems Science & Engineering, ETH Zürich, Basel 4058, Switzerland; Swiss Institute of Bioinformatics, Lausanne 1015, Switzerland

**Keywords:** lineage trees, gene expression, single-cell RNA sequencing, phylogeny

## Abstract

Gene expression data have been proposed as a natural single-cell lineage marker. Here, we critically examine the feasibility of reconstructing lineage trees from single-cell transcriptomic data using both modeling and empirical data. We first introduce a notion of neutrality for transcriptomic data, and then, under a model for neutral gene expression, establish theoretical bounds for accurate lineage tree reconstruction. Our findings indicate that reconstruction guarantees for even small trees or sub-trees require thousands of independent, neutral traits—a condition that is likely rarely met in practice due to the dominance of non-neutral developmental signals. Furthermore, errors introduced by measurement sampling have the potential to destroy any existing lineage signal. We conclude that gene expression data have limited potential as a natural lineage recorder and should not be used for phylogenetic lineage tree inference without further, rigorous validation.

## Introduction

Uncovering cellular lineage trees is a key goal in developmental biology, and accurately capturing lineage relationships is important for downstream analyses (Stadler et al. 2021; Forrow and Schiebinger 2021). We define a cellular lineage tree as the tree representing the ancestral lineage relationships between the sampled cells which are tips in the tree. To this end, lineage tracing technologies have been developed with the specific goal of mapping out such ancestral histories. Developing lineage tracing technologies – based on either imaging or cellular barcoding approaches—is an active area of research (Askary et al. 2025). However, owing to the expensive and often invasive nature of these techniques, a push toward natural lineage markers has emerged. Natural lineage markers are particularly important for studying human development, but these approaches need to be validated against data from experimental and model systems.

Gene expression data have been proposed as a promising candidate for a natural lineage marker (Kester and Van Oudenaarden 2018; Pan et al. 2023; Eisele et al. 2024), either on its own or in combination with low-resolution barcodes. These data are routinely collected and high dimensional. This high dimensionality is particularly attractive, especially in contrast to the current capacity of CRISPR-based barcode recorders (Askary et al. 2025). Both sources of data have the potential to be used together synergistically, since lineage recorders can accurately record deep splits in the phylogeny (Wang et al. 2023; Mulberry and Stadler 2026), but often lack the amount of recording capacity necessary to resolve closely related cells. By contrast, any lineage signal contained in transcriptomic data (if it exists) would be expected to decay over time, but may be promising for resolving shallow splits after recording capacity is exhausted. Phylogenetic reconstruction methods which combine both data modalities, such as LinTiMat (Zafar et al. 2020) and LinRace (Pan et al. 2023), have subsequently been developed. While such methods increase the resolution of the inferred phylogenies as compared to methods which rely solely on barcodes, a lack of ground truth data makes it difficult to assess whether or not the putative relationships indeed reflect the true lineage history.

Early research established the heritability of epigenetic markers—a key regulator of gene expression (Bonasio et al. 2010; Fitz-James and Cavalli 2022). This was followed by research focused on establishing the heritability, or “memory”, of gene expression levels itself in experimental studies (Suter et al. 2011; Phillips et al. 2019; Shaffer et al. 2020), with some recent work having success at identifying small lineage clusters of 1–3 generations from certain subsets of genes (Eisele et al. 2024). Recent research suggests that clonal memory from, for example, DNA methylation persists much longer than from gene expression (Li et al. 2023; Chen et al. 2025).

In principle, all transcriptomic and epigenetic data sources suffer from common issues: notably, the corruption of the stochastic lineage signal by a deterministic developmental signal, and possible convergent evolution (Packer et al. 2019), which may be driven in part by a shared micro-environment. Gene expression in particular suffers from additional complications and is particularly noisy, due to, for example, cell-cycle effects, technical noise and sampling (Carter and Zhao 2021). However, even if we can accurately account for this noise, we expect that expression across the genome is driven by a complex interplay between the transition diagram, lineage tree, and the micro-environment. Disentangling these effects has proven to be challenging.

Given a lack of ground-truth data, modeling is one tool we can turn to in order to investigate questions regarding phylogenetic information content of transcriptomic or epigenetic data. Here, we first examine a recently published data set where both high-resolution lineage tracing data and transcriptomic data are available, and show that there is limited phylogenetic signal in these data, even when attempting to correct for cell-state effects. We then develop a theory for phylogenetic reconstruction from transcriptomic data, and show how, even in best-case scenarios, reconstruction can be erroneous.

## Materials and methods

### Case study: mouse gastruloids

We first investigate the potential of using transcriptomic data as a natural lineage recorder using recently published data from Seidel et al. (2026). In this experiment, mouse gastruloid development was recorded using DNA Typewriter (Choi et al. 2022), from which a high-quality lineage tree was inferred using the Bayesian inference package SciPhy (Seidel et al. 2026). Here, we consider the conditional clade distribution (CCD) phylogeny to be close to a “ground-truth” lineage tree (the CCD tree represents a mean tree over the posterior distribution of inferred trees Berling et al. 2025). We analyze a collection of 285 cells, chosen to contain both high quality lineage information from the recorders as well as transcriptomic profiles (with assigned cell types based on Pijuan-Sala et al. 2019). We will compare phylogenies reconstructed from the transcriptomic data to the phylogeny inferred from the lineage-tracing data.

We reconstruct 6 phylogenies from different subsets of cells: (a) using all 285 cells, (b) choosing a particularly well-resolved clade from the SciPhy CCD phylogeny. This clade contains 14 cells across multiple cell types. (c) Four subsets all composed of a single Pijuan cell type.

**Fig. 1.**
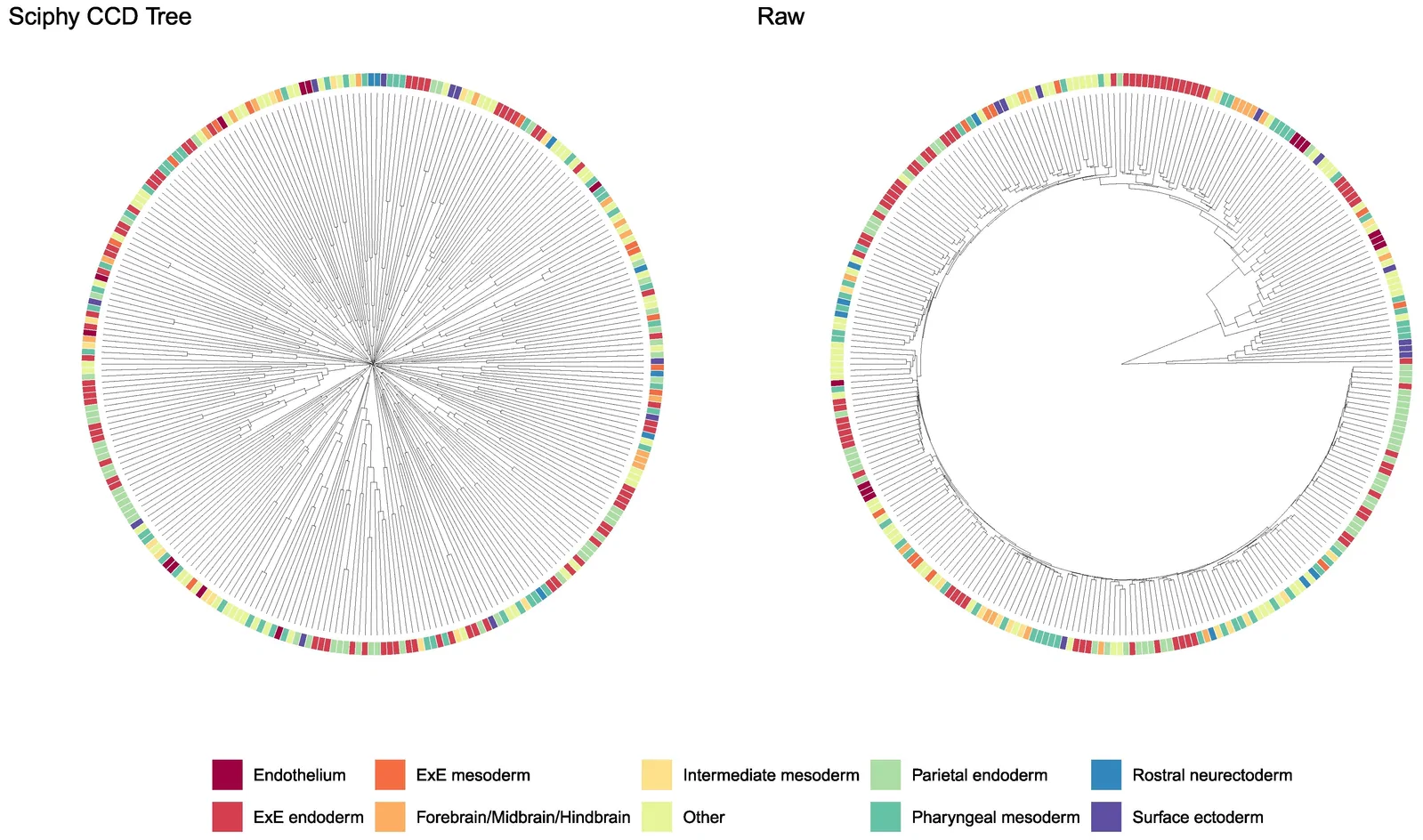
(Left) SciPhy CCD tree with 284 tips annotated with the most common detected cell types. (Right) UPGMA tree build from the raw counts. The resulting trees from normalized data are shown in Supplementary Fig. S3. Tree visualization is from the package *ggtree* (Xu et al. 2022).

**Fig. 2.**
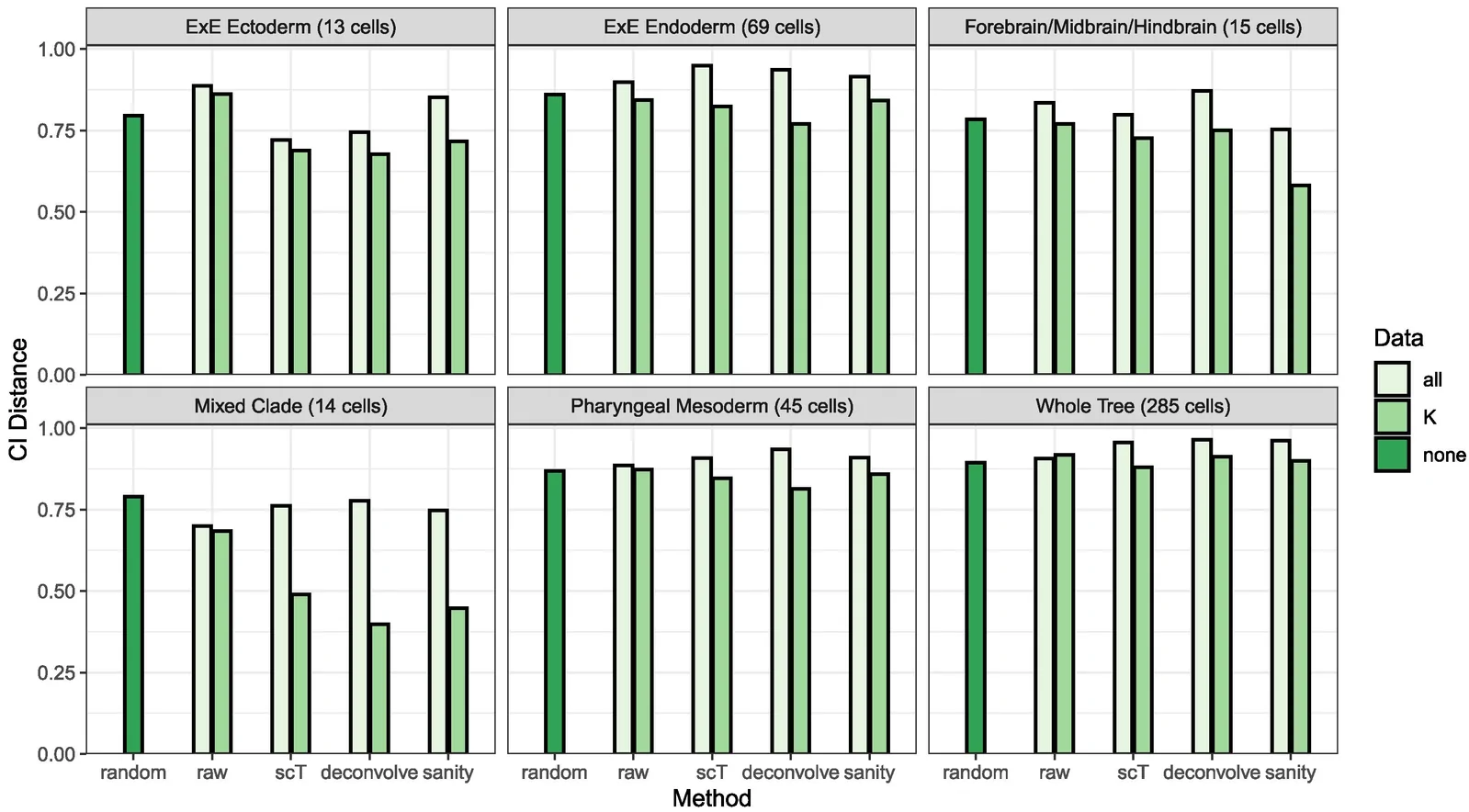
Normalized tree distances between reconstructed trees and corresponding Sciphy CCD phylogeny. We use the raw counts and also compare to two different normalization approaches: scTransform from Seurat (Hafemeister and Satija 2019), Sanity from Breda et al. (2021), and Deconvolve from scran (Lun et al. 2016). The trees generated from transcriptomic data (using both all genes and the fitted *K* genes) are also compared to a random tree (value shown is the median score over 10 samples).

**Fig. 3.**
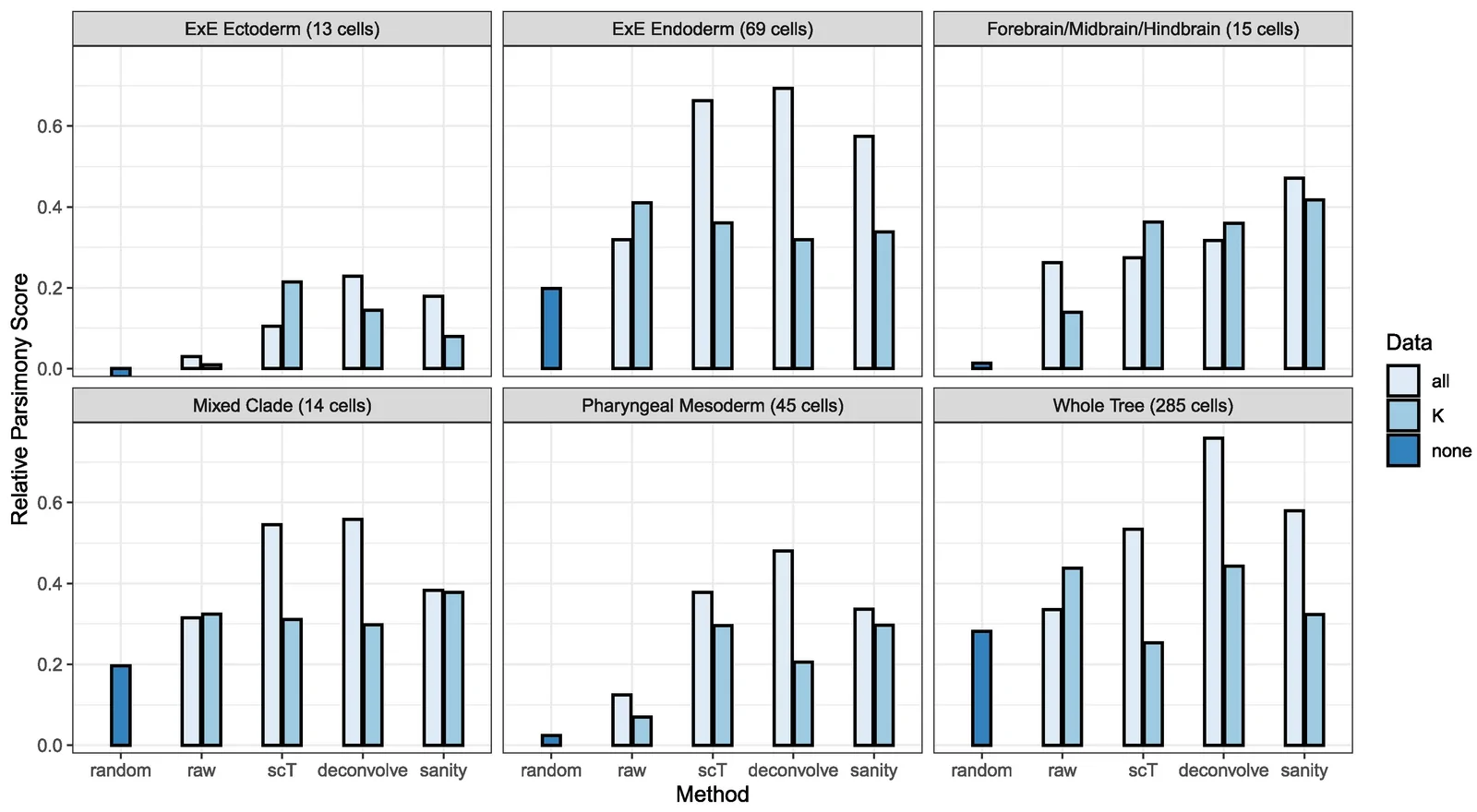
(Inverse) Parsimony scores relative to corresponding SciPhy CCD phylogeny, as computed using the Typewriter barcodes (see Methods). A higher score indicates a less parsimonious phylogeny. Scores are computed for each normalization method, using both all genes and the fitted *K* genes, and further compared against a random tree (median score over 10 samples).

For each subset of cells, we build a proposed phylogeny from the transcriptomic data using the UPGMA algorithm (as shown in Fig. 1 and Supplementary Fig. S3). These putative lineage relationships are then compared to those inferred by the SciPhy CCD phylogeny by comparing the phylogenetic information content (based on the Clustering Information distance introduced by Smith 2020) of each tree (Fig. 2). In the following, we will refer to this distance metric as the CI distance. However, the barcoding data are nonetheless expected to exhibit significant uncertainty with respect to tree reconstruction (Mulberry and Stadler 2026). Therefore, in addition to comparing phylogenetic distances directly, we also compute a parsimony score directly using the Typewriter barcodes for each reconstructed tree as shown in Fig. 3. We employ three different normalization methods and also compare to using the raw counts only. We use the methods scTransform from Seurat (Hafemeister and Satija 2019), Sanity from Breda et al. (2021), and Deconvolve from scran (Lun et al. 2016). We furthermore include a random tree which uses no data as input (see Methods).

In addition to using all transcriptomic data for reconstruction, we also search for a subset of genes which may have increased phylogenetic information content. We do so by computing Blomberg’s K statistic (Blomberg et al. 2003) over each gene using the CCD phylogeny based on the lineage-tracing data, and by taking only those with K>0.95. In this way, this subset of genes represents a best-case scenario: any method which attempts to detect high-signal genes without prior information on the lineage would be expected to perform in between using all transcriptomic data or choosing data based on a statistical test for phylogenetic information content, such as the *K* statistic.

Figure 2 shows that there is almost no overlapping phylogenetic signal in the trees inferred using gene expression data as compared to the phylogeny inferred from the Typewriter barcodes. While using the fitted *K* genes (see Supplementary Fig. S1) improves this picture (as expected), there remains little correspondence between the trees, even in the “within-type” trees where we have attempted to account for cell-state effects. The best performing reconstructed tree comes from the 14-cell mixed clade, which has a more recent root than the other trees. In this case, as with nearly all other cases, however, the trees based on transcriptomes have in most cases significantly worse parsimony scores than their SciPhy counterpart (Fig. 3). This trend appears to be true across all normalization methods used, with the tree built from the raw counts often having the best score. Surprisingly, the reconstructed trees rarely beat the random score; this is likely due to their highly ladderized nature (Supplementary Fig. S3). Overall, these results suggest that there may be limited potential to find a lineage signal in these data.

With these results in mind, we develop a theory for reconstructing lineage trees based on scRNAseq measurements, and use this theory to show how, even in the most optimistic scenarios, we expect there to be limited lineage signal in single-cell transcriptomes, and gene expression data in particular.

### Model

We model the evolution of gene expression according to an Ornstein–Uhlenbeck (OU) process along a cell lineage tree (as done in, for example, Stuart and McKenna 2025). We distinguish between “neutral” and “developmental” genes: neutral genes will be those which are not associated with any cell type or niche. We allow for the case where a neutral gene is pulled toward an optimum, the key difference between a neutral and developmental gene is that, with a neutral gene, this optimum is not cell-type dependent but is constant across all cells for a given gene. The strength of selection acting on each neutral gene will, however, affect its phylogenetic information content (Brownian motion represents the best case scenario, whereby these dynamics are driven entirely by random fluctuations).

Our goals are to provide theoretical estimates and/or bounds for lineage reconstruction based on neutrally evolving genes, and to use these results to illustrate fundamental limitations of using transcriptomic data for phylogenetic reconstruction. We focus on neutral genes for two reasons: (1) it is expected that the cell-state signal dominates the phylogenetic signal in developmental genes (see Methods for more details); and (2) they present both the best-case scenario and a clean framework for theory and simulations. For reference, Table 1 gives a list of the key parameters for both the theoretical model and simulation framework(s).

**Table 1.** Table of key parameters for the different generative processes and simulation frameworks. We refer to the parameter *α* as the “strength of selection”, although we note that we are not referring to an evolutionary mechanism, but rather the strength at which the trait is regulated.

Model	Parameter	Explanation
Neutral OU	*α*	strength of selection
	*σ*	sd of expression level
	$\sigma_{\epsilon }$	sd of additive Gaussian noise
	*η*	ratio: $\sigma_{\epsilon }/\sigma$
	*M*	number of independent, neutral traits
True tree	T	true underlying phylogeny
	*n*	number of tips/sampled cells
	ℓ	minimal branch length
Simulated trees	$n_{g}$	number of generations
	*κ*	governs synchronicity of tree
Sampling error	*k*	governs spread of sampling dist.
	$p_{c}$	capture probability

#### Problem set-up

Let the abundance of each transcript in a cell be an independent, continuous trait that is inherited upon division, and changes randomly through time according to an OU process. The traits thus evolve along the single-cell lineage tree with changing dynamics according to the cell type (in the case of a developmental trait). The *phylogenetic reconstruction problem* seeks to invert this process, and to infer the lineage relationships from the values recorded from a sample of cells, all of which are descendent from a common ancestor.

Developmentally-neutral genes evolve under identical OU processes on the lineage tree, in a manner independent of developmental stage or cell state. In this way, expression values will be correlated with the lineage tree. Meanwhile, for the set of developmentally-aligned genes, cell states will be strongly correlated within discrete types. Sister cells of the same type will generally also have a more highly correlated state than nonsister cells, but lineage relationships between cells of different types will typically be erased.

The main idea behind our theoretical results is that neutral traits, unlike developmentally-aligned ones, are unbiased but noisy lineage recorders. Any randomly chosen individual trait is expected to contain low signal; however, given many such traits, we investigate the potential of these data to be used for phylogenetic reconstruction. Specifically, we calculate the probability that the distance matrix computed from *M* neutral traits reflects the true distance relationships.

We now derive the theoretical reconstruction guarantees from *M* independent, neutral traits evolving along a phylogeny, sampled at the same final time-point.

#### Probability of recovering a triplet

Consider an ultrametric phylogeny T with root at t=0 and tips at a final time $t_{f}$. Without loss of generality, we take $t_{f}=1$. Let *a*, *b* be two samples with a most recent common ancestor at time $0<t_{v}<1$. Similarly, let *c* be a sample such that the most recent common ancestor of all three samples is $0<t_{u}<t_{v}$ (i.e. *a* and *b* are more closely related to each other than to *c*). We will use the notation (a,b|c) to describe such a relationship.

Following a similar framework as established in Mulberry and Stadler (2026), we first consider the problem of resolving any arbitrary triplet (a,b|c) in T. That is, given measurements from each of a,b,c, what is the probability—given the underlying model parameters—that we can recover the true topological relationship (a,b|c). The idea behind our derivation is that distance-based methods (such as UPGMA) will recover the topological relationship (a,b|c) provided $D_{a,b}<\min(D_{a,c},D_{b,c})$ (where *D* denotes the measured distance between two samples).

Let $g_{i}$ be a neutral gene. We further assume that each measurement is affected by additive Gaussian noise (ϵ). This models intrinsic noise of the system, but does not directly measure technical sampling noise or drop-outs. Let $X_{a}^{i}$ denote the measured value (at present) of transcript *i* in cell *a*. Then, under this model, we have


$$X_{a}^{i}-X_{b}^{i}∼N\left(0,\frac{\sigma_{i}^{2}}{\alpha_{i}}(1-\exp\;(-2\alpha_{i}(1-t_{v})))+2\sigma_{\epsilon }^{2}\right),$$

where $\sigma_{i}^{2}$ is the variance of the process and $\alpha_{i}$ is the strength of selection acting on the gene. Similarly,


$$X_{a}^{i}-X_{c}^{i}∼N\left(0,\frac{\sigma_{i}^{2}}{\alpha_{i}}(1-\exp\;(-2\alpha_{i}(1-t_{u})))+2\sigma_{\epsilon }^{2}\right),$$

and the same for $X_{b}^{i}-X_{c}^{i}$. Successful reconstruction will therefore rely solely on the fact that $t_{u}>t_{v}$, and the path length from *u* to *v* will be the major determinant of success.

Now suppose we have *M* such traits which all evolve independently along the phylogeny. Then the total measured distance between the two cells is given by


$$D_{a,b}=\sum_{i=1}^{m}|X_{a}^{i}-X_{b}^{i}|.$$

and similarly for $D_{a,c}$ and $D_{b,c}$. Each distance is a folded normal, for example $\left|X_{a}^{i}-X_{b}^{i}\right|$ is a folded normal with mean


$${\tilde{\mu }}_{a,b}^{i}=\sqrt{\frac{2}{\pi }\left(\frac{\sigma_{i}^{2}}{\alpha_{i}}\left(1-\exp\;(-2\alpha_{i}(1-t_{v}))\right)+2\sigma_{\epsilon }^{2}\right)}$$

and variance


$${\left({\tilde{\sigma }}_{a,b}^{i}\right)}^{2}=\left(\frac{\sigma_{i}^{2}}{\alpha_{i}}\left(1-\exp\;(-2\alpha_{i}(1-t_{v}))\right)+2\sigma_{\epsilon }^{2}\right)\left(1-\frac{2}{\pi }\right).$$

Let


$$\mu_{a,b}=\sum_{i}{\tilde{\mu }}_{a,b}^{i},\;\sigma_{a,b}^{2}=\sum_{i}({\tilde{\sigma }}_{a,b}^{i}{)}^{2}.$$

Then the probability of being able to resolve the triplet is given by


$$P(D_{a,c}>D_{a,b})=P(D_{b,c}>D_{a,b})\approx \Phi \left(\frac{\mu_{a,c}-\mu_{a,b}}{\sqrt{\sigma_{a,c}^{2}+\sigma_{a,b}^{2}}}\right),$$

where *Φ* is the cumulative density function of the standard normal, and we have assumed that the distribution of differences $D_{a,c}-D_{a,b}$ is approximately normal, which holds as M→∞. In the simplifying case where all $\sigma_{i}=\sigma$ and $\alpha_{i}=\alpha$, we get


(1) $$P(D_{a,c}>D_{a,b})\approx \Phi \left(\sqrt{\frac{2M}{\pi }}\frac{\left[\sqrt{\frac{\sigma^{2}}{\alpha }g(t_{u})+2\sigma_{\epsilon }^{2}}-\sqrt{\frac{\sigma^{2}}{\alpha }g(t_{v})+2\sigma_{\epsilon }^{2}}\right]}{\sqrt{\left(1-\frac{2}{\pi }\right)\left[\frac{\sigma^{2}}{\alpha }\left(g(t_{u})+g(t_{v})\right)+4\sigma_{\epsilon }^{2}\right]}}\right),$$

where


g(t)=1−exp(−2α(1−t)).

Now introduce a new variable, $\eta =\sigma_{\epsilon }/\sigma$. Then we can rewrite the above expression as


(2) $$P(D_{a,c}>D_{a,b})\approx \Phi \left(\sqrt{\frac{2M}{\pi }}\frac{\left[\sqrt{\frac{1}{\alpha }g(t_{u})+2\eta^{2}}-\sqrt{\frac{1}{\alpha }g(t_{v})+2\eta^{2}}\right]}{\sqrt{\left(1-\frac{2}{\pi }\right)\left[\frac{1}{\alpha }\left(g(t_{u})+g(t_{v})\right)+4\eta^{2}\right]}}\right).$$

In the special case of Brownian motion (α=0), the left hand side of Eq. (2) becomes


(3) $$\Phi \left(\sqrt{\frac{2M}{\pi }}\frac{\left[\sqrt{(1-t_{u})+\eta^{2}}-\sqrt{(1-t_{v})+\eta^{2}}\right]}{\sqrt{\left(1-\frac{2}{\pi }\right)\left[\left(2-t_{u}-t_{v}\right)+2\eta^{2}\right]}}\right).$$

We note here that this reconstruction probability does not depend on the parameter *σ* in the absence of noise.

#### Reconstruction guarantees

Let ℓ>0 be a bound on the minimal internal branch length in T (i.e. there are no polytomies). The most difficult to resolve triplet in a tree with branch lengths bounded by ℓ will occur when both $t_{u}$ and $t_{u}-t_{v}$ are minimal. We can use Eq. (2) to (approximately) bound the probability that any given triplet in the tree is resolved. Let $p^{*}(\ell )=\min_{T}P(D_{a,c}>D_{a,b})$, which occurs when $t_{u}=t_{v}-t_{u}=\ell$. Then, the probability that any triplet in the tree is exactly resolvable is at least approximately


(4) $$1-2(1-p^{*}(\ell )).$$

We can use a similar procedure to bound the total tree reconstruction accuracy over all triplets, as shown in Supplementary material S1. We here measure accuracy as being the probability that the tree topology is perfectly reconstructed. We use this quantity to estimate the minimal number of traits necessary to guarantee a specified level of accuracy.

## Results

### Reconstruction guarantees for neutral traits

**Fig. 4.**
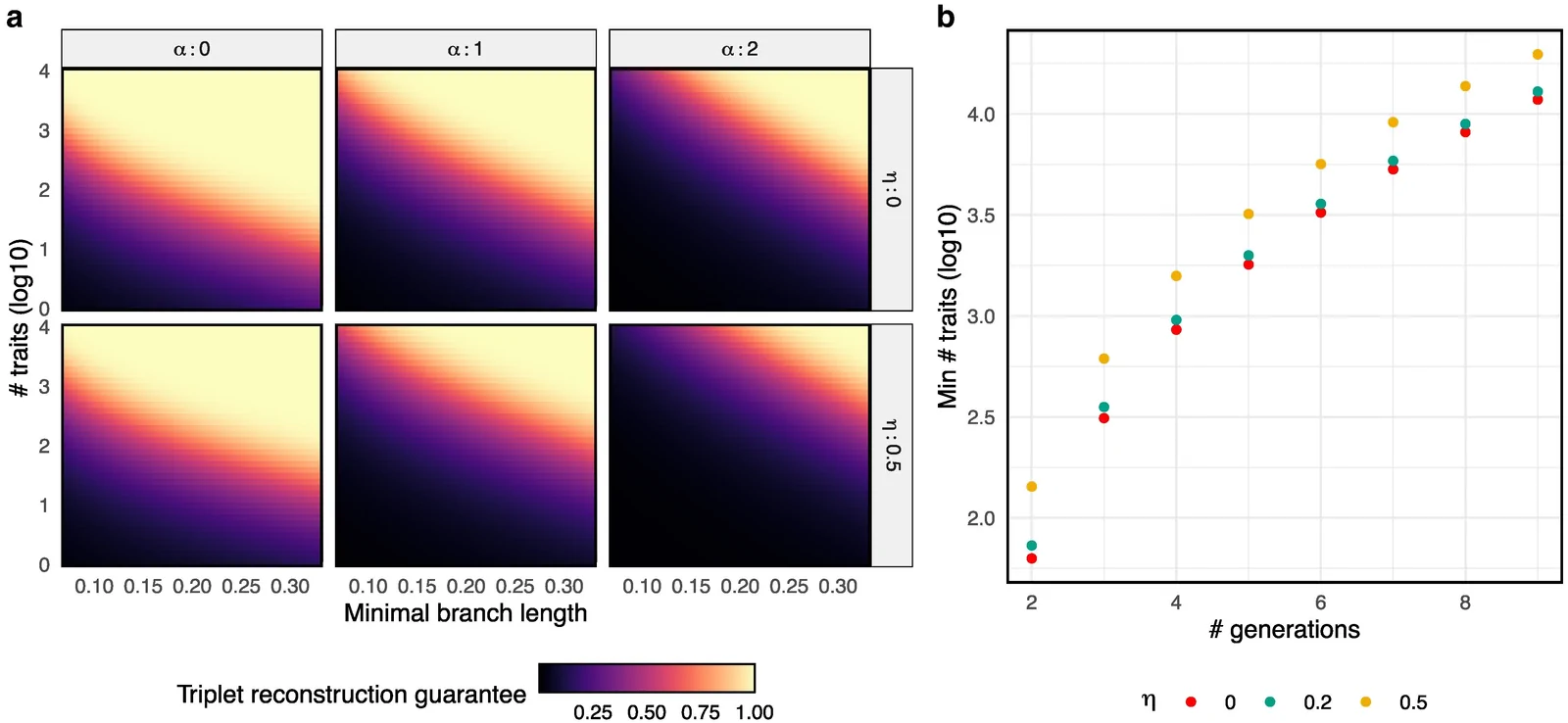
(a) Illustration of the bound on the probability of resolving a single triplet under the neutral OU model in the tree given by Eq. (4). This probability is shown as a function of the number of independent neutral traits *M*, minimal branch length ℓ, the strength of selection *α*, and the noise ratio *η*. (b) Minimum number of independent traits required to reach a theoretical accuracy of at least 95% on a fully-sampled synchronous tree with the given number of generations under Brownian motion (α=0), given by Thm 1 in Supplementary material S1.

While the bound on triplet reconstruction in Eq. (4) does not provide a rigorous bound on the total tree reconstruction, we find that it provides good intuition for where tree reconstruction is likely to be accurate or not. This bound is visualized over a range of parameters in Fig. 4. We see that, in the best-case scenario where α=θ=0, for an “easy” triplet (ℓ≈0.3), approximately hundreds of independent traits are necessary to guarantee the accurate resolution of this basic structure. Decreasing ℓ to 0.1, this number increases to approximately thousands of traits. As expected, the number of traits required grows significantly as we increase both *α* and *η*.

We next investigate the accuracy of the full tree reconstruction problem. Unlike in the case of developmental genes (see Methods), we see that in the big data limit (M→∞), the probability of exact reconstruction tends to 1 across all parameter regimes (provided ℓ>0). Hence, neutral genes have the potential to be used for phylogenetic reconstruction. We furthermore show the minimum number of traits required to guarantee 95% accuracy of a synchronous tree over a given number of generations, according to Thm. 1 (Supplementary material S1). A fully-sampled synchronous tree is one with uniform internal branch lengths; the resulting tree will have $2^{n_{g}}$ tips with internal branch lengths $\frac{1}{n_{g}+1}<\ell \leq \frac{1}{n_{g}}$. We observe in Fig. 4b how the number of independent, neutral traits required to guarantee a theoretical accuracy of 95% quickly becomes infeasible as either the number of generations or the strength of selection increase.

**Fig. 5.**
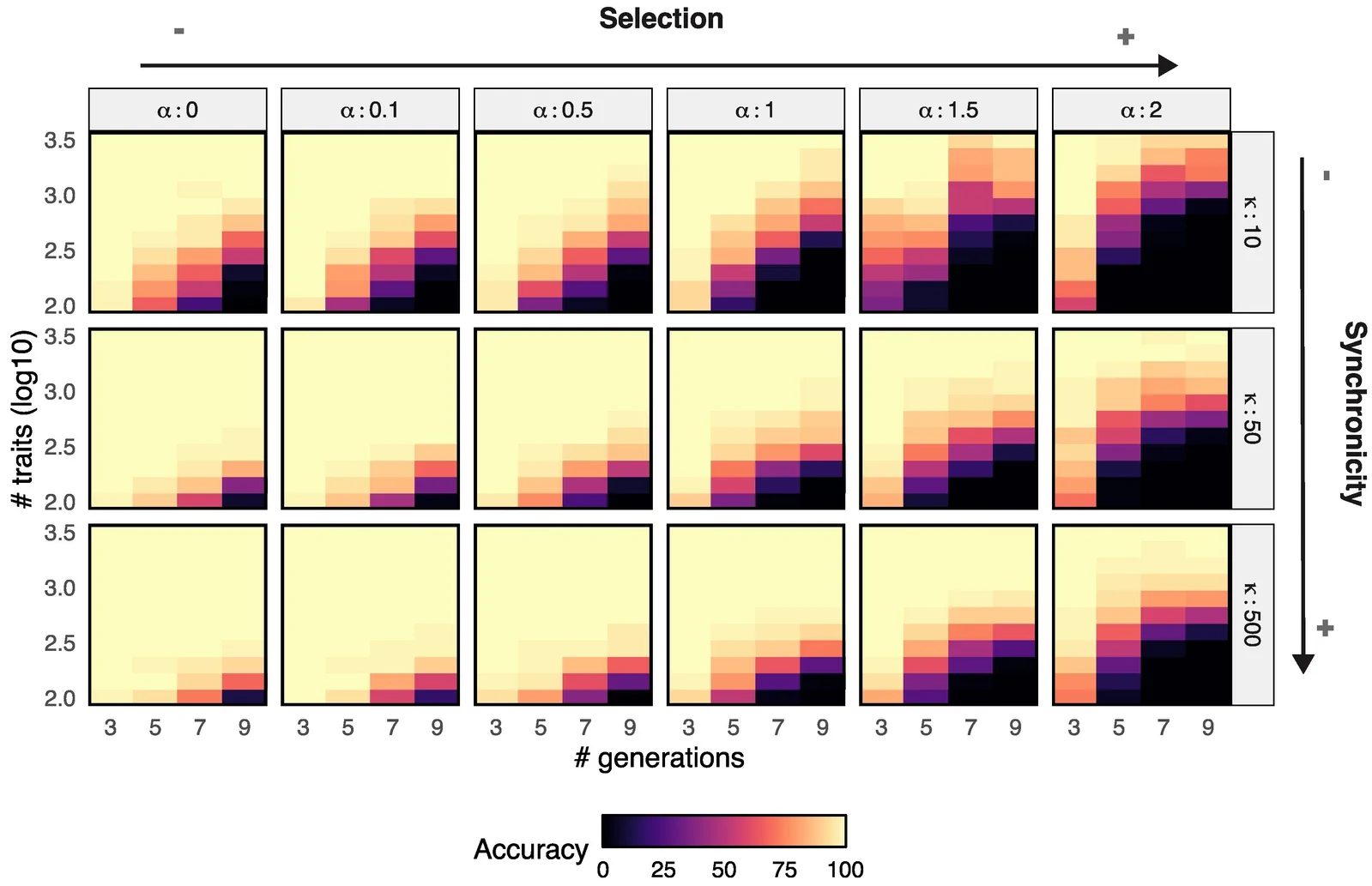
Simulations under identical, neutral OU processes with no errors (η=0). Here *κ* denotes the parameter of the distribution governing the tree generating process. Higher *κ* means more synchronous (see Methods for more details). For each tree, we simulate the model 50 times on the true tree, and compute the accuracy as the proportion of reconstructed trees which correspond to the true tree topology. We repeat this procedure over 10 ground-truth trees for each parameter set, and record the minimum observed accuracy.

While our theoretical bounds provide useful intuition about this problem, such bounds are not always indicative of the actual reconstruction accuracy and are limited to simple scenarios. It is clear from our simulation results below that this bound is not tight—particularly over synchronous trees and as *n* grows. Indeed, when we compare our theoretical results to simulated results (Fig. 5), we find that the theoretical bound on the full tree reconstruction problem overestimates the number of traits required by approx. an order of magnitude. Therefore, we supplement our theoretical results with simulations.

### Simulated results

For each simulation, we first simulate a ground-truth phylogeny, and then simulate an OU process on the tree. We then build a phylogeny from the simulated data (at the tips) using the UPGMA algorithm and compare the resulting tree topologies. We give more details on our simulations in the Methods. We focus here on synchronous trees, as this is both a relevant case for cell lineages (Mulberry et al. 2026) and presents a best-case scenario: the binary tree on a given number of tips with the longest internal branches will be synchronous.

In Fig. 5, we show simulation results where there are no measurement or intrinsic errors. As expected, reconstruction accuracy increases as the trees become more synchronous. In particular, these results suggest that small-scale lineages should be resolved by hundred-to-thousands of independent, neutral traits provided the strength of selection is not too strong. To further illustrate these results, we show the variation over different *σ*’s in Supplementary Fig. S2. As expected from our theory, there is little variation in accuracy over different trees with similar minimal branch lengths. Additionally, provided there are no noise or error terms, the standard deviation of the OU process does not affect the accuracy. Our theoretical results suggest that number of traits required to achieve a given accuracy scales exponentially with the parameter *α*, a trend which appears to be captured by these simulations (Supplementary Fig. S4).

We now move to simulate a more realistic error model. Our model is similar to that of Breda et al. (2021), except that we model continuous expression data instead of discrete UMIs. We simulate data as before, but now model sampling noise explicitly. That is, we take the simulated true value *x* and model the observed, noisy data as $\hat{x}∼\Gamma (\theta (x),k)$ for some shape *k*, and scale $\theta (x)=p_{c}x/k$ for some value of $p_{c}$. The parameter $p_{c}$ represents a capture probability. With this parametrization, the mean observed value is always $p_{c}x$. As the shape parameter *k* increases, the distribution becomes more peaked and the observed value is expected to become closer to $p_{c}x$.

**Fig. 6.**
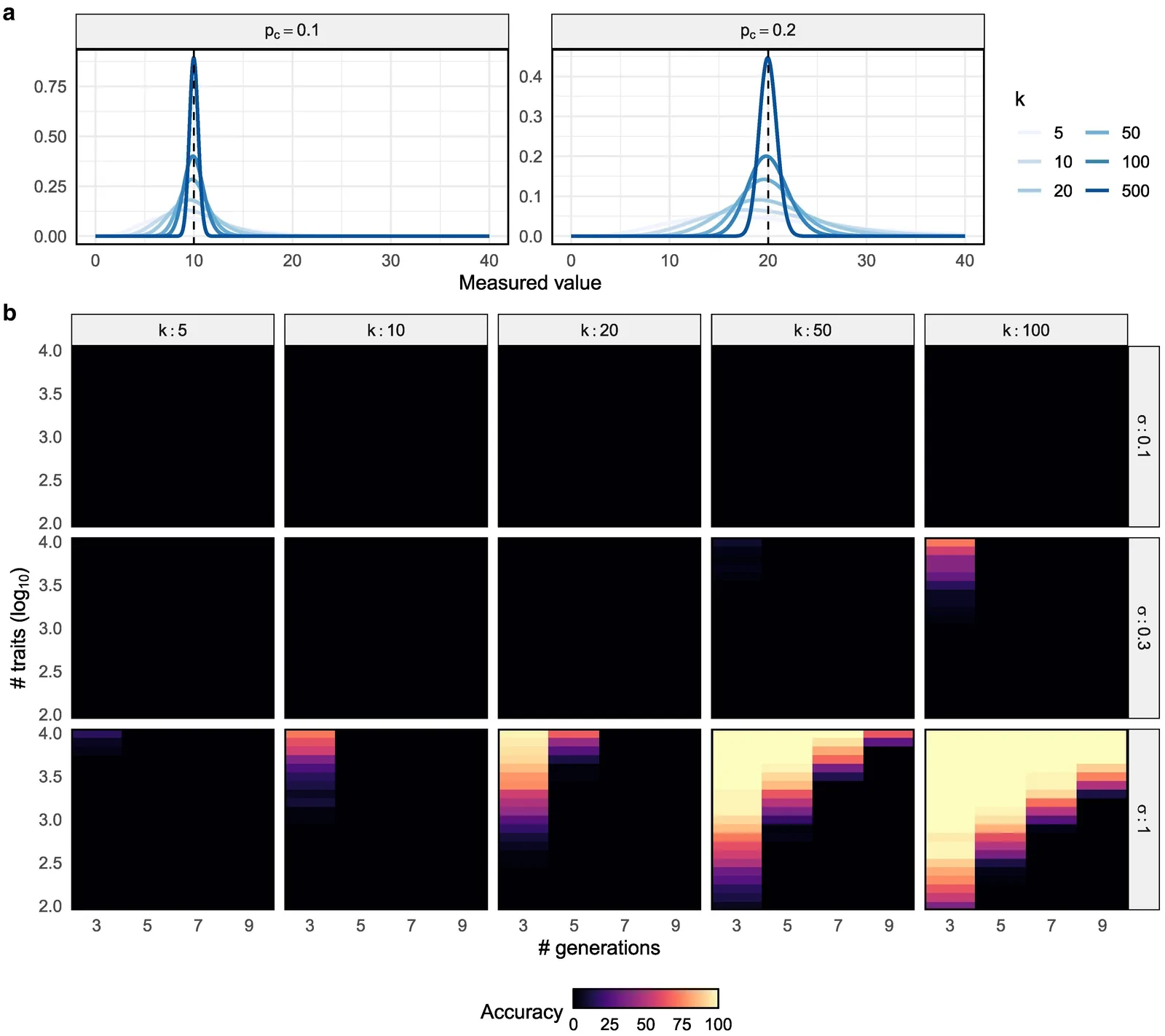
a) Illustration of the error model under simulated parameters. Dashed line shows $p_{c}x$, where *x* is the true underlying value. Here we take, as an example, x=100. b) Simulated accuracy over synchronous trees with $p_{c}=0.1$ (recorded accuracy is minimum over 10 trees). Over each tree, we simulate BM with the error model 50 times. The accuracy is recorded as the proportion of trees which were correctly inferred out of 50. *k* denotes the shape parameter of the gamma distribution used to model the error, while *σ* is the standard deviation of the Brownian process.

In Fig. 6, we show results only for the case of synchronous ground-truth trees and under Brownian motion (α=0) since this is a best case scenario. In general, we find that unless the error model is quite peaked (which is unlikely to be the case for these data), the number of genes required to reconstruct even extremely small lineages quickly becomes unfeasible. These results are also sensitive to the “signal-to-noise” ratio: since we introduce noisy measurement error, accurate reconstruction is only possible if the underlying data have accumulated a sufficient amount of (neutral) variation. While most datasets lack the ability to fit such models directly, recent attempts at fitting similar models suggest that *σ* values may be quite low in practice (Stuart and McKenna 2025).

**Fig. 7.**
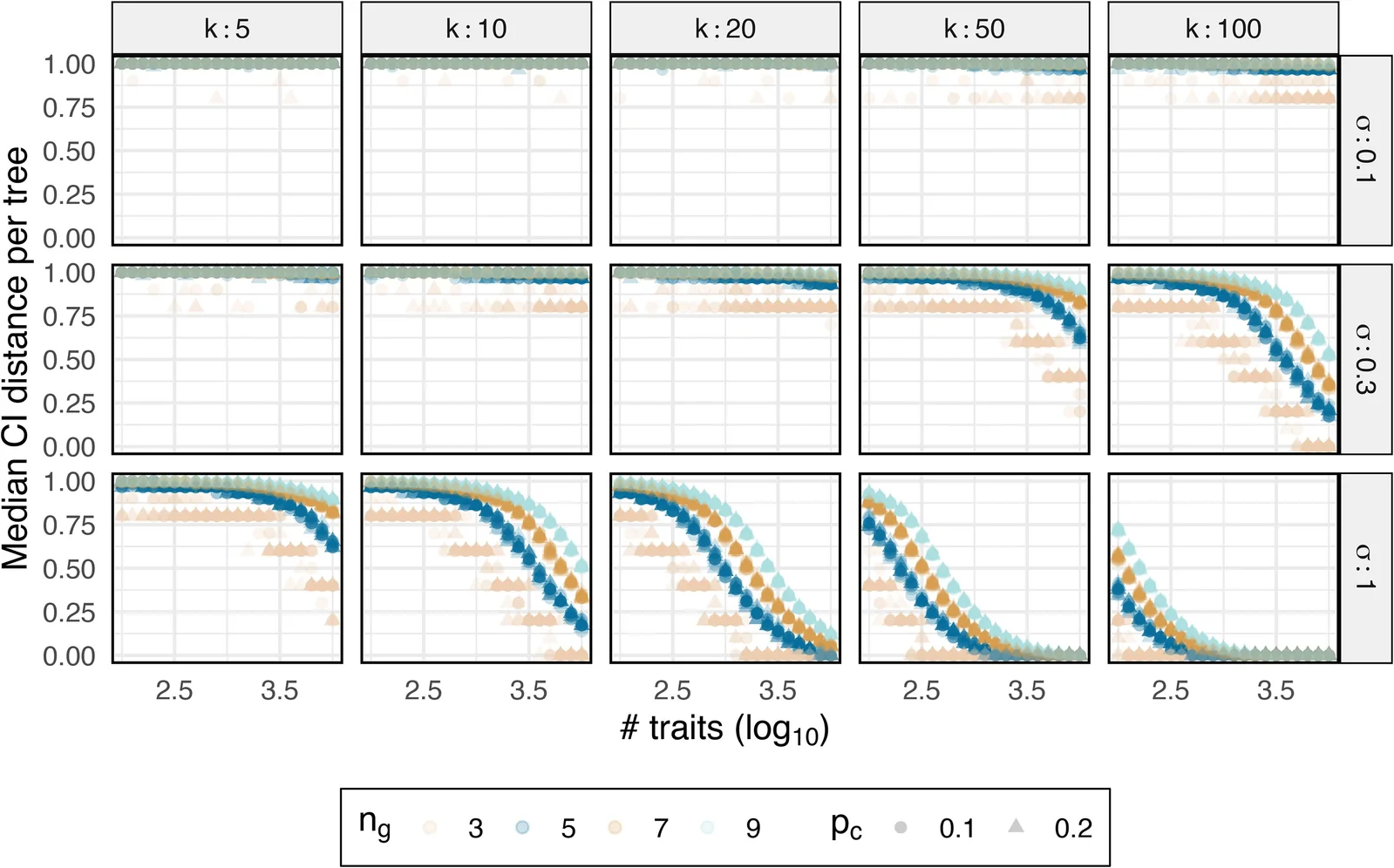
Shows median, normalized CI distances over 50 simulations for each tree (10 trees total). Over each tree, we simulate BM with the error model. For each simulation, the CI distance from the inferred tree to the true tree is recorded.

As a separate metric to the reconstruction accuracy, in Fig. 7 we also measure the distance between the reconstructed and true trees based on the amount of shared phylogenetic information (Smith 2020). We simulate under realistic values of the capture probability (AlJanahi et al. 2018), however we find that the value of $p_{c}$ does not significantly affect the accuracy (Fig. 7). This is likely a consequence of our continuous model; if we were to instead model discrete counts, with a significant probability of generating zero counts, our results would be even more pessimistic than shown here.

## Discussion

We have here argued that transcriptomic data may have only limited potential as a natural lineage marker. Our results here are pessimistic, and suggest that transcriptomic data should not be used to characterize even small lineage trees without further validation and careful quantification of error. Even in relatively optimistic scenarios, accurate reconstruction requires thousands of independent, neutral traits—a condition that may be unlikely to be met in practice. While, based on both theory and simulations, neutral genes could theoretically have sufficient phylogenetic signal, this signal is extremely delicate and easily perturbed by noise, especially the type of sampling noise that is likely to be characteristic of these data. By contrast, the potential for engineered barcodes to resolve lineage trees is much greater (Mulberry and Stadler 2026). Validation would need to come in the form of datasets which contain both the transcriptome and high-quality lineage tracing. This analysis supports previous empirical observations that there is limited signal in gene expression data across different model systems (Packer et al. 2019; Li et al. 2023), and suggests that tools which attempt to cluster lineages based on these data (Eisele et al. 2024) have limited ability to capture detailed phylogenetic relationships.

Our approach here is based on a strict set-up: we use transcriptomic data alone and reconstruct trees using a simplistic distance-based method. While strict, this set-up allows us to investigate the theoretical phylogenetic signal in these data. If these data would be sufficient for accurate reconstruction in our set-up, more sophisticated reconstruction methods which take into account, for example, the generative mechanism of the data and/or combine multiple data modalities would likely be provide even more reliable reconstructions. However, where our results indicate a total lack of signal or conflicting signal, we would expect that even more sophisticated methods would struggle to accurately resolve lineages. Based on our investigations, for any analysis anticipating to use transcriptomic data to inform phylogeny, it is important that the employed methods are able to (1) establish neutrality so as to not introduce conflicting signal, and (2) incorporate the considerable noise and uncertainty inherent to these data.

It would be ideal to be able to connect this theory directly with data. Unfortunately, most currently available data sets (such as the one explored here) tend to be too noisy to be able to fit models such as OU processes directly, due to uncertainty in both the underlying lineage and cell-state trees, as well as extremely sparse transcriptomic measurements. Therefore, we have tried to model either the best-case scenario, or simulate over a wide range of parameters wherever possible. In light of such remaining concerns and lack of ground-truth data, our goal here is not to say that transcriptomic data never contains phylogenetic signal, merely to point out that such claims should be carefully criticized and validated.

## Additional methods

### Data analysis

Raw and processed data for the gastruloid analysis are available under accession number GSE315827. In these data, mouse embryonic stem cells were engineered with DNA Typewriter barcodes capable of six sequential insertions. After approx. 9 d of growth, gastruloids were harvested and processed via scRNAseq (Regalado et al. 2025; Seidel et al. 2026). The Typewriter barcodes were analyzed using the Bayesian phylogenetic inference software, SciPhy. The CCD tree (Fig. 1) was built from approx. 1,000 phylogenies sampled from the posterior tree distribution.

From these raw data, we take different subsets of cells and genes as described in Section “Case study: mouse gastruloids”. For each data set, we compute a distance matrix by calculating the euclidean distance over all counts between each pair of cells. Using this distance matrix, we build a tree using the UPGMA algorithm as implemented in *phytools* (Revell 2012). We repeat this procedure for each subset of genes/cells considered, and for each normalization procedure. On each tree and over each gene, we calculate Blomberg’s K statistic using *phytools* (Revell 2024). The CI distance to the CCD phylogeny was computed using the TreeDistance function from the *TreeDist* package (Smith 2020), which uses the ‘ClusteringInfo’ metric. The CI distance is a generalized RF distance, but still considers only tree topology.

#### Calculating order-aware parsimony scores

The parsimony score is based on typewriter barcodes, which enforce a sequential editing structure (Choi et al. 2022), and so should follow the generative model of these data (Seidel et al. 2026). We calculate the parsimony score by first annotating each tree with the most parsimonious, order-aware internal state for each barcode. The final score is then found by counting the required number of independent substitutions along each branch.

#### Random trees

We furthermore compare our results against a randomly generated tree. These trees are generated under biologically plausible parameters using the R package *scTreeSim* (Mulberry et al. 2026). We set the cell lifetimes to follow a gamma distribution with shape parameter 20 and scale 1, and generate a tree of $2^{15}$ cells which is then sampled and pruned down to the relevant tree size. Tip labels are randomly assigned.

### Developmentally-aligned traits

In general, we expect the cell-cell distances calculated from non-neutral traits to be dominated by cell type differences. For example, suppose we have two annotated cell types and a type-colored tree. Assume for this example that the splits correspond to differentiation times. Let $\mu_{1}^{i}$, $\mu_{2}^{i}$ be the long-term means of types 1 and 2, respectively. Then, if we have an asymmetric triplet (a,b|c), where nodes *a* and *c* share type 1, while node *b* has type 2, we have


Xai−Xbi∼N((μ1i−μ2i)(1−exp(−α(1−tv)),σ2ασ2α(1−exp(−2α(1−tv)))),

while


$$X_{a}^{i}-X_{c}^{i}∼N\left(0,\frac{\sigma^{2}}{\alpha }(1-\exp\;(-2\alpha (1-t_{u})))\right).$$

Therefore, even in the big-data limit (M→∞), such traits could not be guaranteed to have phylogenetic signal.

### Simulations

Trees were again simulated using *scTreeSim*. For each simulation, the distribution of cell ‘lifetimes’ is specified as a gamma distribution with mean lifetime ℓ and shape parameter *κ*. As *κ* increases, the simulated lifetimes become more tightly centered around the mean value, and the resulting tree becomes more synchronous and balanced. Final time is fixed to $t_{f}=1$. To generate synchronous trees on $n_{g}$ generations, we fix the shape parameter to be κ=500 and set the scale parameter to be $1/(\kappa n_{g})$. We do not sample the trees since the main parameter governing the accuracy is the minimum internal branch length; the number of tips itself has a lesser effect.

An OU process is simulated on the true tree using *ape* (Paradis and Schliep 2019). Unlike the real process, for analytical tractability we do not enforce a boundary at 0. Therefore, to keep our simulations true to the model, we initialize with a high expression level, $r_{0}$ (typically, $r_{0}=1,000$). The long-term mean is drawn randomly $\mu =r_{0}+r$, where r∼Unif(10,1,000). Our results are not sensitive to *μ* as long as it is large enough. The final state is either the absolute value of the true simulated value, or sampled from the true value under the error model.

From the end-point data, we build a distance matrix and then construct a UPGMA tree. We calculate the accuracy as being the proportion of simulations which result in exact reconstruction.

## Supplementary Material

iyag187_Supplementary_Data

## Data Availability

Raw and processed data for the gastruloid analysis are available under accession number GSE315827. Code to reproduce analyses can be found at: github.com/nmulberry/gene-expression-trees. Supplemental material available at GENETICS online.
