# Supplementary material for: Should we build single-cell lineage trees from gene expression data?
Source: Genetics. 2026 Jul 18;234(1):iyag187. doi: 10.1093/genetics/iyag187 (PMC13535251; doi:10.1093/genetics/iyag187)
Supplement: Supplementary file 1 [file iyag187_supplementary_data.pdf]

Supplementary material: Should we build single-cell lineage trees from gene expression data?

Nicola Mulberry[1,2] and Tanja Stadler[1,2]

[1]Department of Biosystems Science and Engineering, ETH Zürich, Basel, Switzerland
[2]Swiss Institute of Bioinformatics, Lausanne, Switzerland

S1 Bound on tree reconstruction

We now consider a bound on the probability of resolving every triplet in the tree using Eq. 1. Let $\mathcal{T}$ denote the true tree, and $\hat{\mathcal{T}}$ denote the tree which is built from the observed distance matrix (for example, using some statistically consistent distance-based method such as UPGMA). Taking a union bound over all triplets, we get:

$$P(\hat{\mathcal{T}} = \mathcal{T}) \geq 1 - 2\binom{n}{3}(1 - \min_{\mathcal{T}} P(D_{a,c} > D_{a,b})), \tag{1}$$

where n is the number of sampled cells. Note that this considers only the topology of the two trees (and not branch lengths).

We now note that Eq. 1 is minimized when the distance $|(u, v)|$ is minimal, and for t_u minimal (deep split). Suppose we want to guarantee a reconstruction accuracy of at least A. Accuracy is here defined as the probability that the reconstructed tree has the exact same topology as the true tree. Taking $t_u = \ell$, $t_v = 2\ell$ and re-arranging for M gives us the following result:

Theorem 1 *Given a true underlying phylogeny $\mathcal{T}$ on n tips with minimal branch length $\ell > 0$, the tree reconstructed from M independent traits evolving under identical OU processes is guaranteed to have an accuracy of at least A provided*

$$M \gtrsim (\Phi^{-1}(A_n))^2 \frac{(\pi/2 - 1)\left[\frac{1}{\alpha}\left(g(\ell) + g(2\ell)\right) + 4\eta^2\right]}{\left(\left[\sqrt{\frac{1}{\alpha}g(\ell) + 2\eta^2} - \sqrt{\frac{1}{\alpha}g(2\ell) + 2\eta^2}\right]\right)^2}.$$

where α is the strength of selection of the OU process, $\eta = \sigma/\sigma_\epsilon$ (the ratio of the standard deviations of expression and noise), and

$$A_n = 1 - \frac{1 - A}{2\binom{n}{3}}, \text{ and } g(t) = 1 - \exp(-2\alpha(1 - t)).$$

We can use Theorem 1 to investigate the minimum number of independent traits needed to guarantee a theoretical accuracy of at least 95% using Theorem 1. In practice, we find that this greatly overestimates the required number of traits, especially on synchronous trees. To improve this bound, it is likely necessary to find an approach which does not require bounding over all triplets, which we leave for future work.

S2 Additional figures

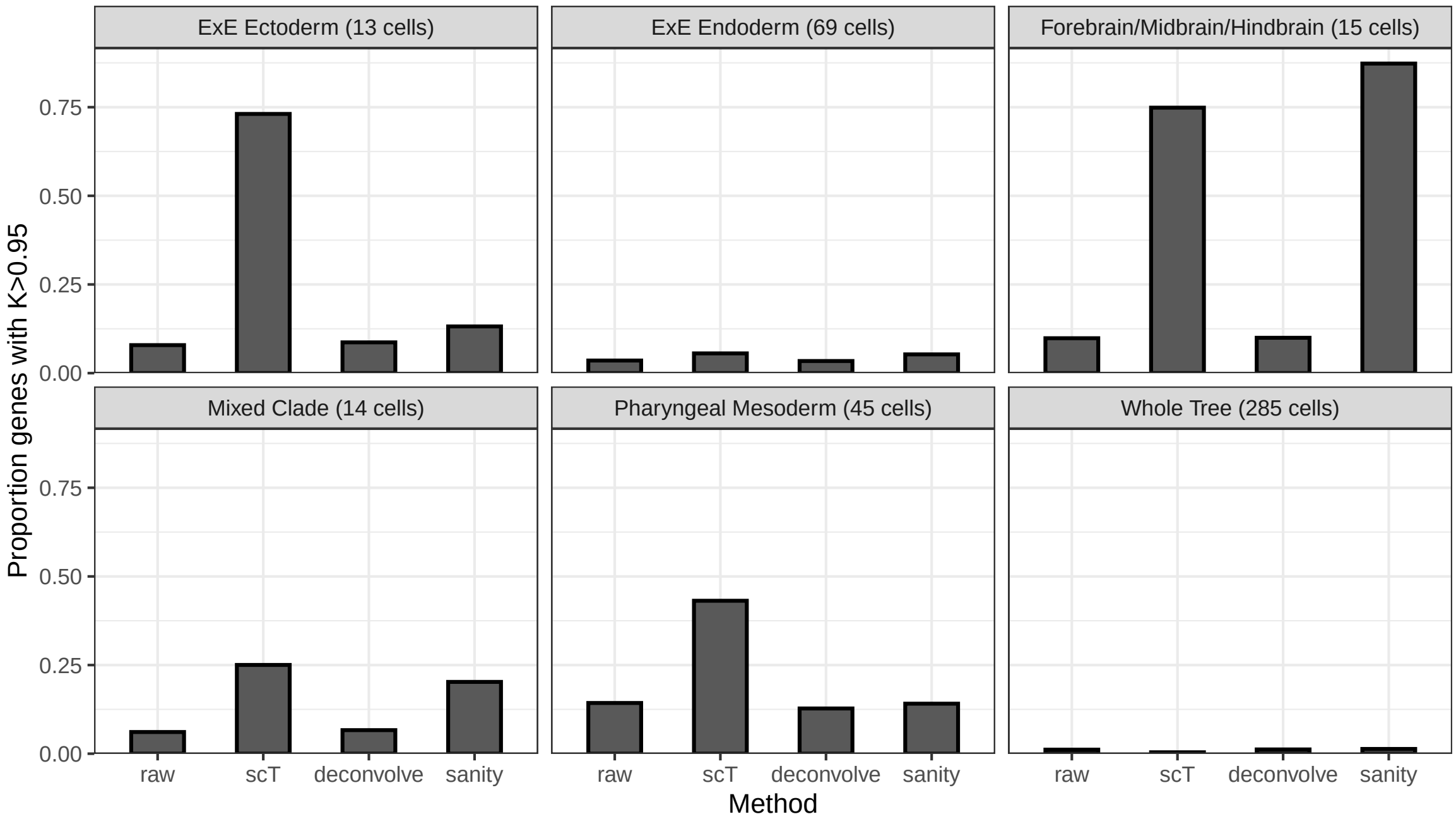

Figure S1: Proportion of genes with $K > 0.95$ for each run shown in Figures 2 and 3.

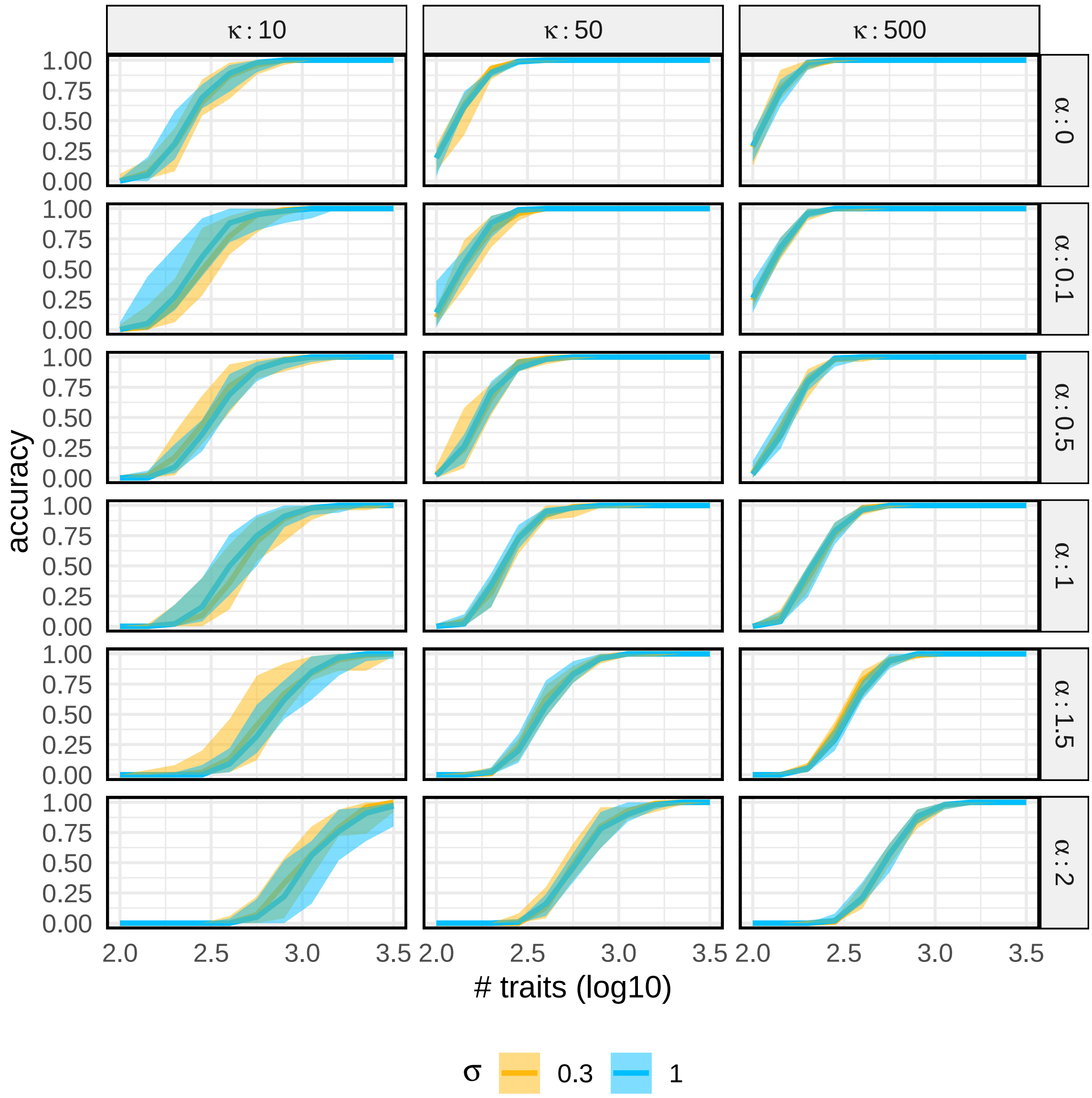

Figure S2: Our theory predicts that, in the absence of any noise or error terms, the standard deviation of the OU process does not affect the accuracy. We see this reflected also in our simulation results. Simulations are as in Figure 5, over 9-generations. The parameter κ governs the synchronicity of the underlying phylogeny, with higher κ indicating a more synchronous tree.

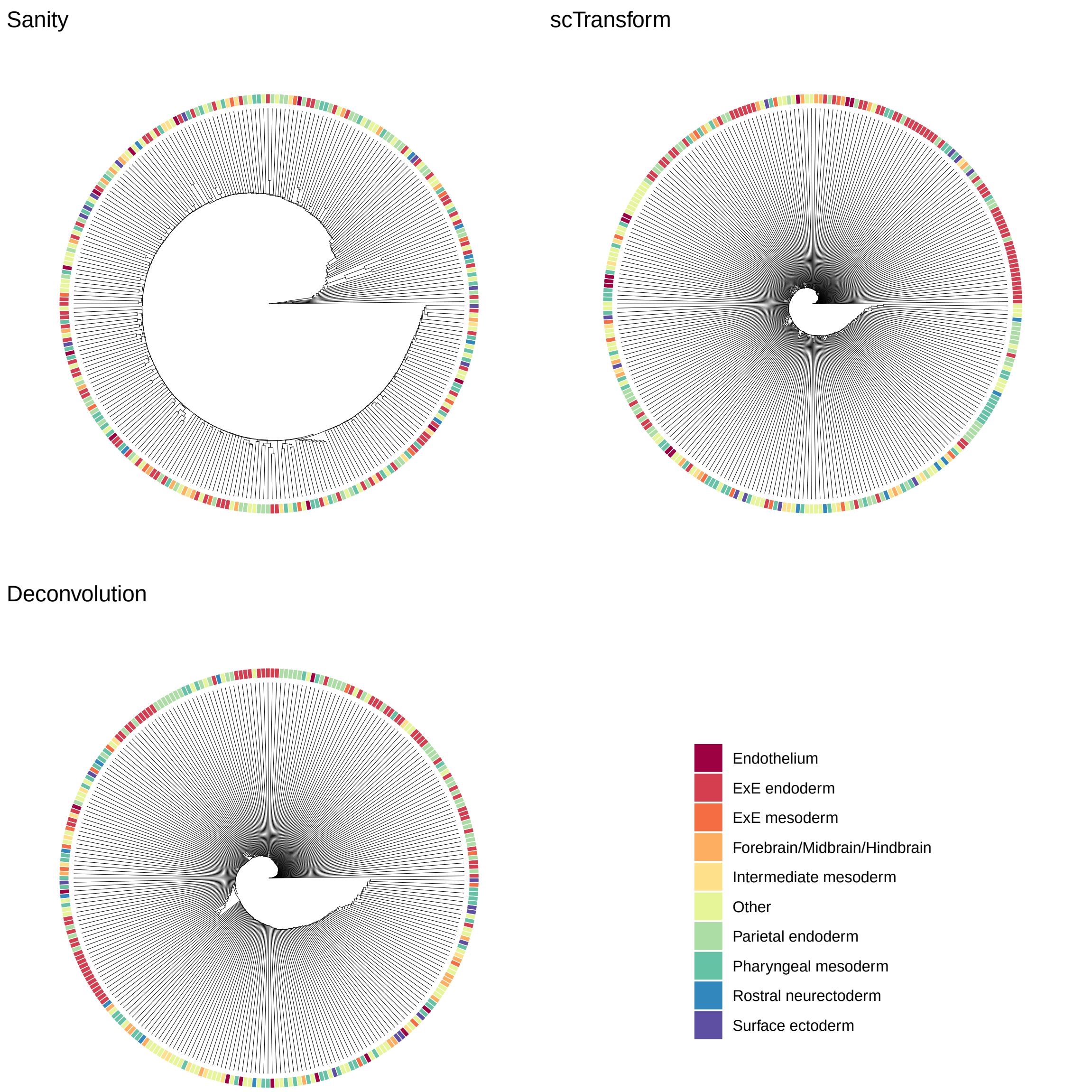

Figure S3: UPGMA trees built from applying different normalization methods to the raw gene expression counts. Normalization methods used are Sanity from Breda et al. [1], scTransform from the Seurat package [2], and the deconvolution method from scran [3]. Tips are annotated with their inferred cell types (showing only the 9 most common).

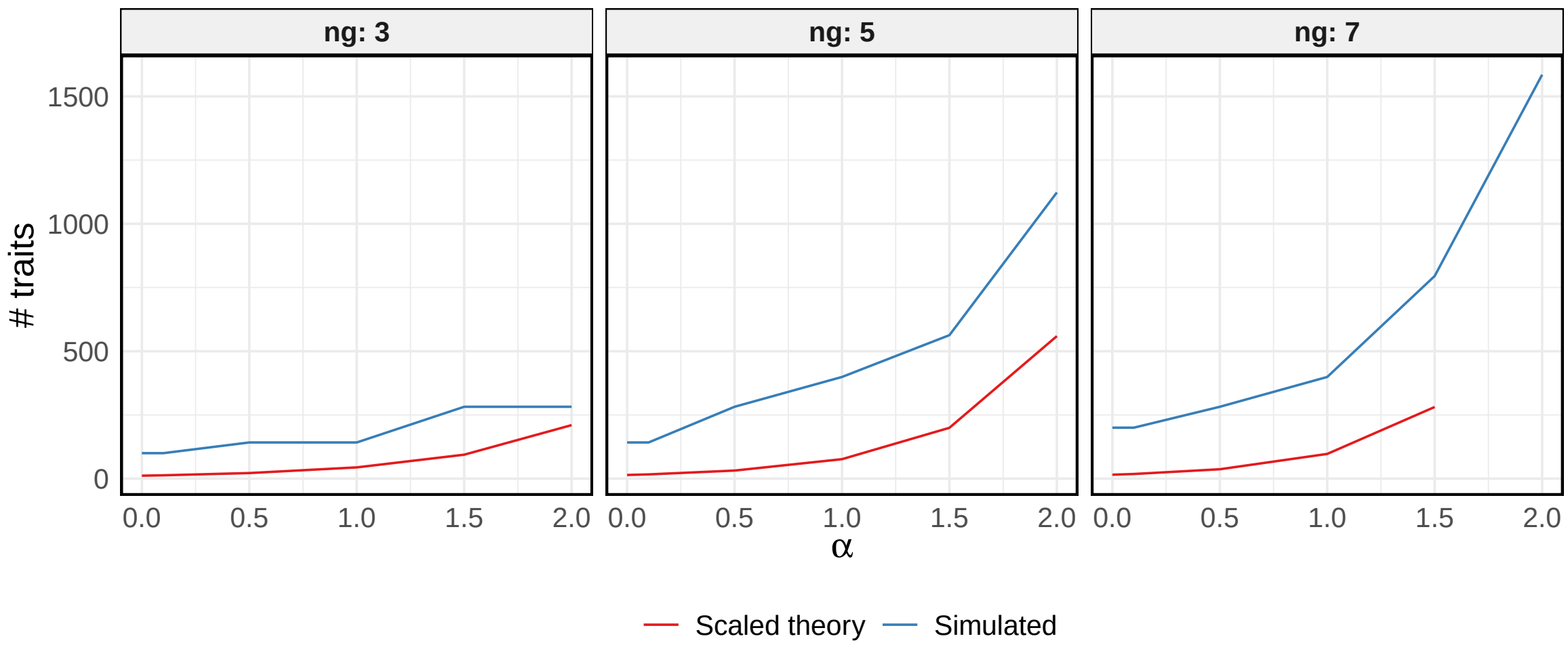

Figure S4: Comparison of scaling with respect to α. (Blue) Simulated results for the minimum number of traits required to achieve an accuracy of 0.95 over synchronous trees ($\kappa = 500$) for the given number of generations, ng. (Red) Theoretical results given by applying Thm.1, scaled by ng^3.

References

[1] Jérémie Breda, Mihaela Zavolan, and Erik van Nimwegen. Bayesian inference of gene expression states from single-cell RNA-seq data. *Nature Biotechnology*, 39(8): 1008–1016, 2021.

[2] Christoph Hafemeister and Rahul Satija. Normalization and variance stabilization of single-cell RNA-seq data using regularized negative binomial regression. *Genome biology*, 20(1):296, 2019.

[3] Aaron T L. Lun, Karsten Bach, and John C Marioni. Pooling across cells to normalize single-cell rna sequencing data with many zero counts. *Genome biology*, 17(1):75, 2016.
